# New Scalable Sulfur Cathode Containing Specifically Designed Polysulfide Adsorbing Materials

**DOI:** 10.3390/ma17040856

**Published:** 2024-02-12

**Authors:** Artur M. Suzanowicz, Bianca Turner, Thulitha M. Abeywickrama, Hao Lin, Dana Alramahi, Carlo U. Segre, Braja K. Mandal

**Affiliations:** 1 Department of Chemistry, Illinois Institute of Technology, Chicago, IL 60616, USAbiancat@andrew.cmu.edu (B.T.);; 2 Department of Physics & CSRRI, Illinois Institute of Technology, Chicago, IL 60616, USA; segre@iit.edu

**Keywords:** lithium–sulfur batteries, bulk sulfur cathode synthesis, titanium dioxide, polypyrrole, polyaniline, carbon nanotubes, lithium polysulfides

## Abstract

Because of its considerable theoretical specific capacity and energy density, lithium–sulfur battery technology holds great potential to replace lithium-ion battery technology. However, a versatile, low-cost, and easily scalable bulk synthesis method is essential for translating bench-level development to large-scale production. This paper reports the design and synthesis of a new scalable sulfur cathode, S@CNT/PANI/PPyNT/TiO_2_ (BTX). The rationally chosen cathode components suppress the migration of polysulfide intermediates via chemical interactions, enhance redox kinetics, and provide electrical conductivity to sulfur, rendering outstanding long-term cycling performance and strong initial specific capacity in terms of electrochemical performance. This cathode’s cell demonstrated an initial specific capacity of 740 mA h g^−1^ at 0.2 C (with a capacity decay rate of 0.08% per cycle after 450 cycles).

## 1. Introduction

Due to their exceptional theoretical specific capacity of more than five times that of lithium-ion batteries (LIBs) and other transition metal oxide-based cathodes (1675 vs. 300 mA h g^−1^) and energy density (2600 vs. 265 Wh kg^−1^), lithium–sulfur batteries (LSBs) have been viewed as a viable replacement for lithium-ion batteries (LIBs) [1,2,3,4]. Most notably, sulfur is inexpensive, widely available, and safe for the environment, making it a perfect substitute for rare-earth and hazardous metals like cobalt, included in most LIBs [5]. However, due to a few significant problems, LSBs have not yet been produced on a large scale. First, the infamous lithium polysulfide (LiPS) shuttle of electrolyte soluble higher-order polysulfide intermediates, Li_2_S_n_ (4 ≤ n ≤ 8) [6], causes irreversible losses of active material as the long-chain polysulfides react with lithium metal through a disproportionation reaction and reduce to insoluble Li_2_S/Li_2_S_2_ particles [7,8]. Second, the significant volume expansion (~80%) of Li_2_S during discharge (d_sulfur_ = 2.03 g/cm^3^ vs. d_Li2S_ = 1.66 g/cm^3^) causes the disintegration of the cathode structure, leading to poor electrochemical performance and capacity decay [9,10,11]. Third, the insulating properties of both elemental sulfur and Li_2_S result in an insufficient depth of discharge [1,2,3,4].

To address some of these challenges, numerous cathode designs involving electrically conductive hosts made of carbon, such as porous carbon [12,13,14,15,16,17], carbon nanotubes [18,19,20,21], and graphene [22,23,24,25,26,27,28,29], have been developed. However, additional polar materials, such as TiO_2_, MnO_2_, graphitic carbon nitride (*g*-C_3_N_4_), N/P doped carbon, and conducting polymers, are needed to trap lithium polysulfides (LiPSs) because carbon alone is nonpolar. Carbon has no permanent charge difference and cannot stop the slow migration of LiPSs [30,31]. The interaction and binding energies between different functional groups containing carbon, nitrogen, sulfur, fluorine, chlorine, and other elements were studied theoretically by Cui and colleagues using density functional theory. Carbon and halogenated groups have the lowest binding energies with LiPSs, whereas oxygen, nitrogen, and sulfur groups have the greatest. This strong chemical reaction can limit sulfur particles in the cathode and significantly lessen the polysulfide shuttle effect. However, a major limitation of this technique is that most of these polar materials have poorer electrical conductivities than carbon, which limits fast electron transit and full utilization of sulfur. As a result, for robust chemical trapping of LiPSs during cycling and electrical conductivity, an appropriate cathode design incorporating sulfur, polar material, and carbon is necessary [32].

Electrically conducting polymers (ECPs) offer new possibilities for prolonging the cycle life of lithium–sulfur batteries (LSBs) because of their low density, electrical conductivity, mechanical structure, ease of production and scale-up, and low cost [33]. Some of the most well-known examples are polypyrrole (PPy) and polyaniline (PANI) [34]. PPy is redox-active, electronically conducting, and has characteristics of an organic semiconductor once it is stabilized/doped with counter-ions. The polymer is also polysulfide adsorbing, not to mention biocompatible and ecologically stable [35]. John et al. modified a sulfur cathode by making composites with polypyrrole and graphene. This PPyS-based material displayed an initial capacity of 1248 mA h g^−1^ with a reversible capacity of 800 mA h g^−1^ after 100 cycles at 0.1 C (capacity retention 64%) [36].

With a theoretical specific capacity of 294 mA h g^−1^, PANI can take part in the electrode’s redox reaction with elemental S via the S-S bond [37]. In addition to improving the transport of electrons and ions, PANI is essential for cyclability, particularly for buffering the volumetric changes of electrode materials. Furthermore, PANI can be included directly in LSBs as an electroactive component due to its strong affinity for S and polysulfides via the quinonoid imine (−N=) of its quinone ring [38]. In order to slow the capacity decline in lithium–sulfur batteries, Li et al. developed a unique structure where sulfur was loaded on polyaniline-graphene nanoribbons (PANI-GNRs). The sulfur-PANI-GNR cell had stable reversible specific discharge, decreasing only 9% over 374 cycles at 0.4 C [39].

Our team created a novel, scalable composite sulfur cathode material for this study named BTX (S@CNT/PANI/PPyNT/TiO_2_) via simple and rapid synthesis. Unlike the PANI-GNRs prepared by Li et al., the sulfur-loaded composite was created for the first time using lithium–sulfur technology through the in situ polymerization of aniline in the presence of carbon nanotubes (CNTs) and polypyrrole nanotubes (PPyNT), with concurrent production of titanium dioxide (TiO_2_) nanoparticles. Specifically, a mixture of elemental sulfur, PANI, PPyNTs, CNTs, and TiO_2_ was autoclaved to create BTX, resulting in an electrically conducting framework for S mechanically strengthened by CNTs and covalently bonded S. During the two-stage heating process, a portion of sulfur vulcanized, forming cross-linked interchain disulfide bonds with polyaniline [40]. The remaining sulfur diffused into the freshly formed polymer networks and the PANI-PPyNTs-CNTs-TiO_2_ hierarchical network. In brief, the role of each component in this cathode structure is as follows. The redox-active PANI and PPyNTs have characteristics of organic semiconductors once they are stabilized with counter-ions, not to mention they are biocompatible and ecologically stable [33]. Additionally, PANI, PPyNTs, and TiO_2_ capture the soluble lithium polysulfide intermediates through potent chemical and physical adsorption effects. CNTs enhance the cathode structure’s electrical conductivity, and PANI is crucial in maintaining the structure’s cohesiveness by creating a network of sulfur crosslinks.

## 2. Experimental Section

### 2.1. Materials

Aniline (99+%), titanium n-butoxide (98%), ammonium persulfate (98.0%), ethanol (99%), dichloromethane (99%), iron(III) chloride hexahydrate (98%), and sublimed sulfur (~100 mesh, 99.5%) were purchased from Alfa Aesar. Pyrrole (98%) was purchased from Sigma-Aldrich, St. Louis, MO, USA. Methyl orange (0.1% *w*/*v*) was purchased from Ricca. Sulfuric acid (36 M) was purchased from Fisher Chemical, Chicago, IL, USA. Multiwalled carbon nanotubes were purchased from Cheaptubes.com (5 May 2021), where the vendor listed the electrical conductivity as >100 S/cm. All reagents were used as received without further purification.

### 2.2. Preparation of PPyNT

In a standard procedure, 200 mL of a 5 mM methyl orange aqueous solution was used to dissolve 10 mmol iron(III) chloride hexahydrate, creating an aggregate [41]. After cooling the liquid to 5 °C, 0.7 mL of pyrrole was added to it dropwise over the course of two hours. Filtration was used to separate the PPy precipitate, which was refined using Soxhlet extraction in acetone until the extracts were colorless. Ethanol was then used to wash and dry the extracts in a vacuum at 40 °C.

### 2.3. Preparation of CNT/PANI/PPyNT/TiO_2_

Aniline was polymerized in the presence of CNTs and PPyNTs. The synthesis was performed by first homogenizing PPyNTs, CNTs, and aniline in a small vial with a few drops of H_2_SO_4_. The mixture was then cooled to −4 °C, and a DCM solution of titanium n-butoxide (TBOT) was added, followed by APS serving as the oxidizing agent. The mixture was further homogenized in a vortex mill, leading to the polymerization of aniline and the formation of TiO_2_ nanoparticles. The mixture was filtered, washed with H_2_O and EtOH, and dried at 80 °C overnight.

### 2.4. Preparation of S@CNT/PANI/PPyNT/TiO_2_ Composite

The S@CNT/PANI/PPyNT/TiO_2_ (BTX) composite was prepared using the melt-diffusion technique. Elemental sulfur was mixed by grinding with CNT/PANI/PPyNT/TiO_2_ in a weight ratio of 7:3. The mixture was then transferred into a Teflon-lined autoclave, blanketed with Ar and heated 1st at 165 °C for 4 h and 2nd at 280 °C for 12h, and then naturally cooled to room temperature. The resulting composite became BTX, where it was partially vulcanized and infiltrated with sulfur. It is noteworthy that S vapor is infiltrated into the host at 165 °C. At 280 °C, S forms crosslinking bridges between sections of PANI chains. We did investigate the sulfur infiltration/crosslinking process at different temperatures; however, we found that the protocol presented here is most optimal.

### 2.5. Characterization

Pictures of the sample structures were taken to verify their identities using a scanning electron microscope (SEM) (Phenom Pro-X). A small quantity of each sample was spread out using a piece of copper tape as a substrate. A 5 keV accelerating voltage was used for imaging and spot analysis in secondary electron mode. Using Ni-filtered Cu Kα radiation, powder-XRD patterns of S@CNT/PANI/PPyNT/TiO2 were produced in Bragg–Brentano geometry using a Bruker D2 Phaser. Data were obtained with a LynxEye linear position sensitive detector from 10° to 90° 2θ degrees. A counting duration of 1.5 s and a step width of 0.01° of 2θ were employed. The sulfur content of the cathode samples was measured using thermogravimetric analysis (Mettler Toledo TGA2) by heating the material from room temperature to 600 °C at a heating rate of 10 °C/min under the continuous flow of nitrogen gas.

### 2.6. Electrochemical Measurements

A mixture of 80% active material, 10% Super P carbon, and 10% polyvinylidene fluoride (PVDF) in N-methyl pyrrolidone (NMP) solvent created viscous slurries. The mixture was agitated in a vortex mill overnight. Al foil was used as the current collector, and the cathode composite film was cast on it. The coated foil was dried at 60 °C overnight. After drying, the cathodes were cut into discs measuring 7/16” (1.11 cm). The weight of the active component (sulfur) in the composite film varied between 0.95 and 1.5 mg/cm^2^. Li metal was employed as the anode, and CR2032-type coin cells were utilized to assess the electrochemical performance. A commercially available lithium metal disc with a thickness near 50 μm was employed as the anode. The excess lithium is about five times that of the amount of sulfur. This is the minimum amount of lithium we can take if we want to keep both cathode and anode sizes equal. A polypropylene-based Celgard separator porous membrane served as the separator. The electrolyte was composed of 25 μL of 1 M lithium bis (trifluoromethane sulfonyl)imide (LiTFSI) in a 1:1 *v*/*v* solution of 1,2-dimethoxyethane (DME) and 1,3-dioxolane (DOL), with a 2 weight percent LiNO_3_ addition. The electrolyte to sulfur ratio was ~1 µL/mg, which is slightly less than what is reported in most studies. A glove box with argon gas was used to make the cells under less than 1 ppm O_2_. With a scan rate of 0.0001 V/s, cyclic voltammetry (CV) was conducted. A range of 100,000 Hz to 0.01 Hz was covered by electrochemical impedance spectroscopy (EIS). Using a Neware battery testing device, the galvanostatic charge–discharge (GCD) investigation was carried out in the potential range of 1.7–2.8 V vs. Li^+^/Li. The specific capacity of the cell was calculated based on the amount of sulfur present in the cathode while counting the theoretical capacity of sulfur at 1675 mAh/g.

## 3. Results and Discussion

**Figure 1:**
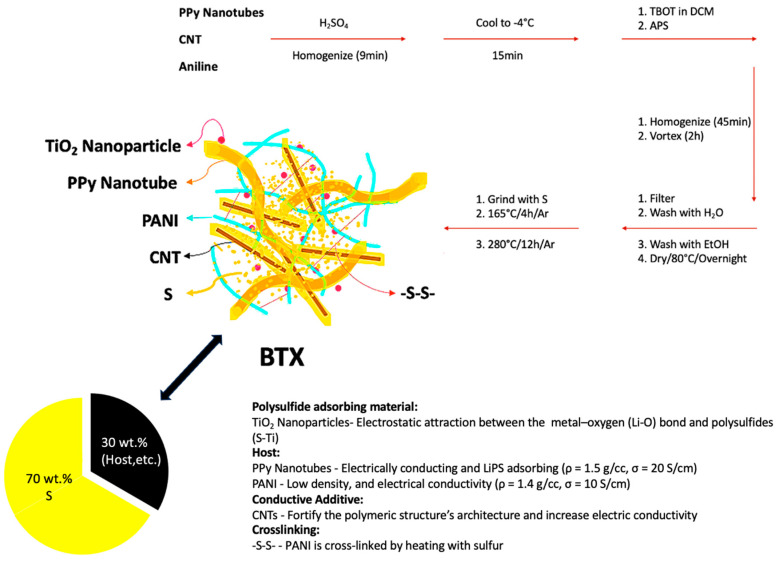
Schematic diagram of the synthesis of BTX cathode composite.

Figure 1 depicts the schematic diagram of the synthetic strategy for the BTX composite cathode material, as described in the experimental section. In this study, several formulations were evaluated and the most optimal condition is presented in the paper. The cathode was comprised of the following fractions: ~70% S and ~30% host, etc. The 30% was comprised of the following optimal fractions: 15.4% TiO_2_NP, 15.4% PPyNT, 53.8% PANI, and 15.4% CNT. The key benefit of this synthetic scheme is that this cathode material does not require nano-synthesis followed by tedious isolation of products. The ingredients can be scaled up simply by increasing their amounts. In contrast to Li et al., our cathode design is more practical and scalable. We used commercially available CNTs instead of graphene nanoribbons that require a lengthy process and the use of corrosive chemicals to make. The CNTs were able to fortify the polymeric structure’s architecture and increase electric conductivity. Because of their distinct nanostructure and exceptional conductivity of up to 100 S/cm, CNTs are superior to conventional carbon materials [42]. CNTs have remarkable mechanical endurance, consistent chemical characteristics, surface roughness in microstructure, and a low coefficient of thermal expansion due to the sp^2^ carbon–carbon bonds.

Additionally, CNTs can construct 3D networks [43]. Furthermore, we synthesized in situ TiO_2_ nanoparticles that significantly assisted polysulfide adsorption. Although the exact nature of the sulfide binding with the metallic oxide has not yet been clearly explained, data show that both the terminal and bridging S in LiPSs interact with Ti, where electrons are polarized away from S to the electropositive transition metal [44]. Appropriate metal oxide-based electrocatalysts improve performance in terms of specific capacity and long-term cycle stability, as demonstrated by Kim et al. [45]. TGA was performed to verify crosslinking, showing minimal weight loss between 150 and 350 °C after heating at 280 °C vs. complete loss of S after infiltration at 165 °C.

**Figure 2:**
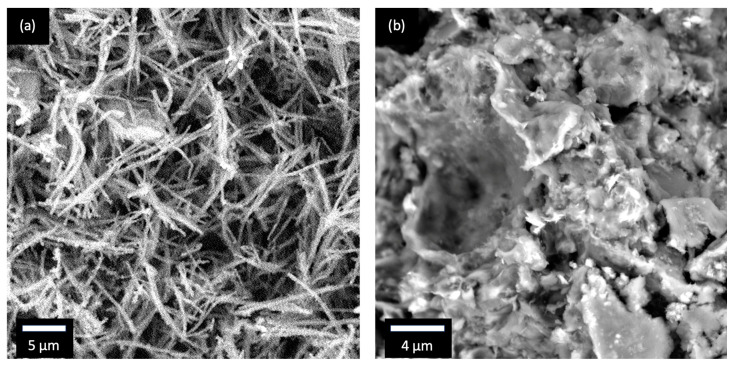
SEM images of (**a**) PPyNT and (**b**) BTX.

Figure 2a shows the morphology of PPyNTs characterized by scanning electron microscopy. The intertwined, 1D nanotubular rods, with an average diameter of ∼100 nm, offer a high aspect ratio and can be filled with sulfur. Figure 2b represents BTX as observed by SEM. The as-synthesized composite showed cluster-like morphology with an average diameter of ∼4 µm and a thickness of ∼100 nm. The ordered PANI serving as host material directly encapsulated all components following two-step heating with PPyNTs visible at the periphery or edges of the clusters. In addition to electrical conductivity, PPy offers the additional benefit of polysulfide adsorption. TiO_2_ was added specifically for that purpose. The PPyNTs are conducting polymers with one-dimensional (1D) fibrous morphology that have several advantages over the typical quasi-spherical shape of particles. In this study, we used this fibrous material as an electrically conducting filler in the BTX cathode composite, in which the percolation threshold is lower than the globular structure, allowing for antistatic and conducting properties even in a low content of added material.

**Figure 3:**
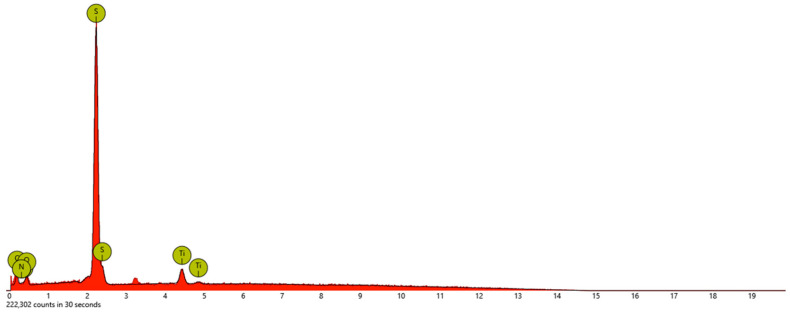
EDS of BTX.

The composition of BTX was further studied with electron dispersive X-ray spectroscopy (EDS). Five distinct sites were scanned for quantitative analysis, and the measured data were averaged. In Figure 3 and Table 1, the presence of S in the sample is represented by the tallest peak in the spectrum, showing 40.69 wt. % followed by C, O, N, and Ti, showing 32.57, 13.46, 6.91, and 6.37 wt. %, respectively. The amount of elemental S is reduced significantly from its initial 7:3 ratio. This was caused by higher temperature heating (280 °C), forcing S crosslinking that was verified by TGA analysis, and further dilution resulting from slurry making.

**Figure 4:**
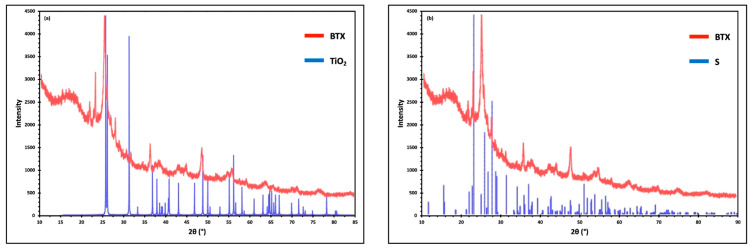
XRD patterns of TiO_2_ (**a**) and S (**b**) with respect to BTX.

Figure 4 shows the XRD patterns of TiO_2_ (a) and S (b) collected from BTX. Each pattern’s prominent peaks matched the sublimed S peaks (ICDD PDF card number 00-064-0585), confirming an orthorhombic crystal structure and the Fddd space group. Since BTX contains TiO_2_, two significant peaks at 26° and 48° showed TiO_2_ (ICDD PDF card number 00-015-0875) in the anatase phase. These peaks are diffracted from (101) and (200) planes, respectively [46].

**Figure 5:**
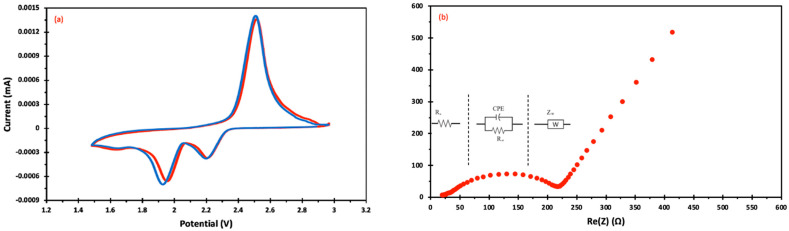
(**a**) Cyclic voltammogram curve of BTX cells for the 2nd (red) and 3rd (blue) cycle; (**b**) electrochemical impedance spectroscopy plot of BTX cells.

By creating CR-2032-coin cells, the electrochemical performance was assessed. The cells comprised BTX (cathode), electrolytes/separators, and metallic lithium (anode). The BTX cathode’s cyclic voltammogram curve for the second and third cycles is displayed in Figure 5a. At a scan rate of 0.1 mV/s, the CV was examined within the potential range of 1.5–3.0 V. Li-S cell CV curves typically exhibit two reduction and one oxidation peaks. Reduction occurs when polysulfides of high and low orders, namely (Li_2_S_n_, 4 ≤ n ≤ 8) and (Li_2_S_2_/Li_2_S), respectively, are produced. Li_2_S_2_/Li_2_S undergoes oxidation when transformed into high-order polysulfides and then back to sulfur [36]. The CV plot of BTX resembles a typical Li-S CV with an oxidation peak at around 2.5 V, indicating delithiation of S during the charging process. The reduction peaks at 2.2 V and 1.9–2.0 V correspond to the discharging process’s conversion from elemental sulfur to long-chain polysulfide (Li_2_S_n_, 4 ≤ n ≤ 8), with a further reduction to Li_2_S in the second peak. As the cycle number increased, the oxidation and reduction peaks increased along with redox currents, indicating enhanced usage of active materials and that more sulfur was engaged in the redox process. However, a shift to lower potential indicated that the materials could rearrange to more electroactive positions.

**Figure 6:**
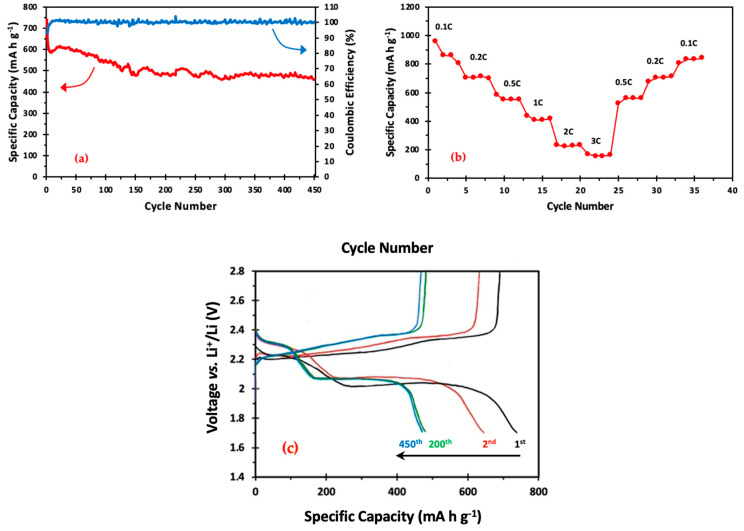
(**a**) Discharge capacity and Coulombic efficiency of BTX cells at 0.2 C; (**b**) rate capabilities of BTX cells at different rates; and (**c**) voltage profiles of the BTX composite electrode at 0.2 C.

The electronic conductivity of the cathode material and the rate of Li^+^ diffusion were evaluated using electrochemical impedance spectroscopy (EIS). Figure 5b shows a typical impedance plot of a BTX cell. It comprises one linear diffusion drift in the low-frequency area and one semicircle in the high- and middle-frequency regions. The creation of a solid film of Li_2_S and Li_2_S_2_ and charge-transfer resistance are associated with the semicircles in the high-frequency (HF) and middle-frequency (MF) regions, respectively [47,48]. For the BTX cell, the HF resistances, R_0_ and R_ct_, were 7.2 Ω and 130 Ω, respectively. An equivalent circuit (Figure 6b) was utilized to fit the EIS plot. In this circuit, CPE stands for the constant phase element, R_0_ is the electrolyte ohmic resistance the semicircle displays in the high-frequency region, R_ct_ is the charge transfer resistance and semicircle diameter, and W_0_ is the Warburg impedance. In the low-frequency range, it is shown by the dotted, sloped line [49,50].

Figure 6a shows the long-term cycling and Coulombic efficiency of BTX at the rate of 0.2 C over 450 cycles. Typically, we run four cells for each cathode. Unless the cell is shorted, the results were within 10%. The presented data represent the best cell. At cycle 1, the cell delivered a good reversible specific discharge capacity of 740 mA h g^−1^; it reduced to 645 mA h g^−1^ and stabilized at cycle 10, where it only degraded by 23% for the remainder of 450 cycles, displaying favorable capacity retention. Furthermore, the Coulombic efficiency was ≥99%, indicating very good electron transfer during charging and discharging. In most LSB research, a notable decline has been noted after the first cycle [51,52,53]. The reason for this quick, irreversible capacity decline is that the stable solid–electrolyte interface (SEI) layer has not fully developed during the first few cycles. Moreover, due to sulfur overflow or agglomeration, certain sulfur particles are partially activated and cannot engage in the lithiation/delithiation reaction. [49]. Not to be overlooked is the cell’s exceptionally good capacity retention from 150 to 450 cycles. (~94%). However, “shuttling” is apparent in cycles 1–150, where the loss of active materials occurs probably due to un-infiltrated sulfur in the cathode structure. However, after 150 cycles, the shuttle effect becomes minimal.

By cycling coin cells via a steady increase in current densities (0.1 C, 0.2 C, 0.5 C, 1 C, 2 C, and 3 C) and then returning to the initial current density of 0.1 C (1 C = 1675 mA g^−1^), the rate performance of the BTX cathode was ascertained. Each cell was tested for four cycles at the specified current density to determine the associated specific discharging capacity after each cycle. Figure 6b displays the step-cycling discharge capacity of the BTX cell for four cycles at each designated current density. The BTX cathode delivered a very high initial specific capacity of 909 mA h g^−1^ at 0.1 C. A steady decrease in the discharge capacity is observed with an increasing cycle rate. BTX did, however, continue to sustain 167 mA h g^−1^ at 3 C. The specific capacity of the cathode recovered to ≥99% of its prior capacity (709 and 706 mA h g^−1^), respectively, when the current density was switched back to 0.2 C.

Figure 6c shows the charge/discharge profiles of the chosen cycles. Every curve shows a large plateau during charging and two more notable plateaus corresponding to the discharging phase. This result aligns with the two significant reduction peaks and one central oxidation peak found by CV testing. Furthermore, the cell’s high stability and capacity retention between 200 and 450 cycles is visible. Moreover, Table 2 shows the performance of other previously reported composites utilizing TiO_2_ and PPy compared to the electrochemical performance of BTX. The BTX cathode material demonstrated favorable capacity and long-term stability at 0.2 C, as indicated by the data reported in Table 2. TiO_2_NP and PPyNT additions to the cathode structure, which offer active sites with a high affinity to anchor polysulfides and shorter diffusion paths for Li^+^ ions, are responsible for this increased stability.

## 4. Conclusions

We have designed and produced a scalable sulfur cathode (S@CNT/PANI/PPyNT/TiO_2_ (BTX) with materials particularly intended to adsorb polysulfide. Discharge–charge profiles revealed that the structure exhibited observable LiPS trapping characteristics, particularly after and beyond 150 cycles. More precisely, a favorable performance at higher charge/discharge rates was made possible by the composite structure’s good sulfur encapsulation and electrical conductivity, and the additional polar materials provide a more effective anchoring of LiPSs, which lessened capacity fading during long-term cycling. This study showed that some of the primary problems in LSBs can be resolved using a highly conductive polymer-based sulfur host that has been improved with metal oxide. After 450 cycles, our composite cathode’s capacity degradation rate of just 0.08% per cycle, with an initial specific capacity of 740 mA h g^−1^ at 0.2 C, was acceptable. This flexible, inexpensive, readily scaled bulk synthesis technique is crucial for transferring bench-level research and development to industrial manufacturing. We believe that further innovative strategies (e.g., the use of conductive titanium nitride (TiN) as polysulfide adsorbing material) can further aid in LiPS trapping and simultaneously improve the electron transport ability, thus increasing the capacity and cycle life of Li-S cells [56].

## Figures and Tables

**Table 1:** Energy dispersive X-ray spectroscopy of BTX. All results are reported based on the weight concentrations of each element.

Sample	Element				
	Sulfur	Carbon	Oxygen	Nitrogen	Titanium
BTX	40.69	32.57	13.46	6.91	6.37

**Table 2:** Comparison between the performance of BTX composite and other scalable LSB cathode materials.

Sample	Current Rate	Initial Capacity (mAh/g)	Reversible Capacity (mAh/g)	Number of Cycles	Reference
S@WLC-CNTs	0.2 C	862	547	300	[54]
SULFUN matrix	0.2 C	970	290	500	[55]
g-KBC/S	0.1 C	1000	740	200	[33]
SPG	0.4 C	673	514	400	[39]
BTX	0.2 C	740	459	450	This study

## Data Availability

Data are contained within the article.
